# Supplementary material for: A potential tool for marine biogeography: eDNA-dominant fish species differ among coastal habitats and by season concordant with gear-based assessments
Source: PLoS One. 2024 Nov 11;19(11):e0313170. doi: 10.1371/journal.pone.0313170 (PMC11554088; doi:10.1371/journal.pone.0313170)
Supplement: S1 Fig — (DOCX) [file pone.0313170.s018.docx]

**
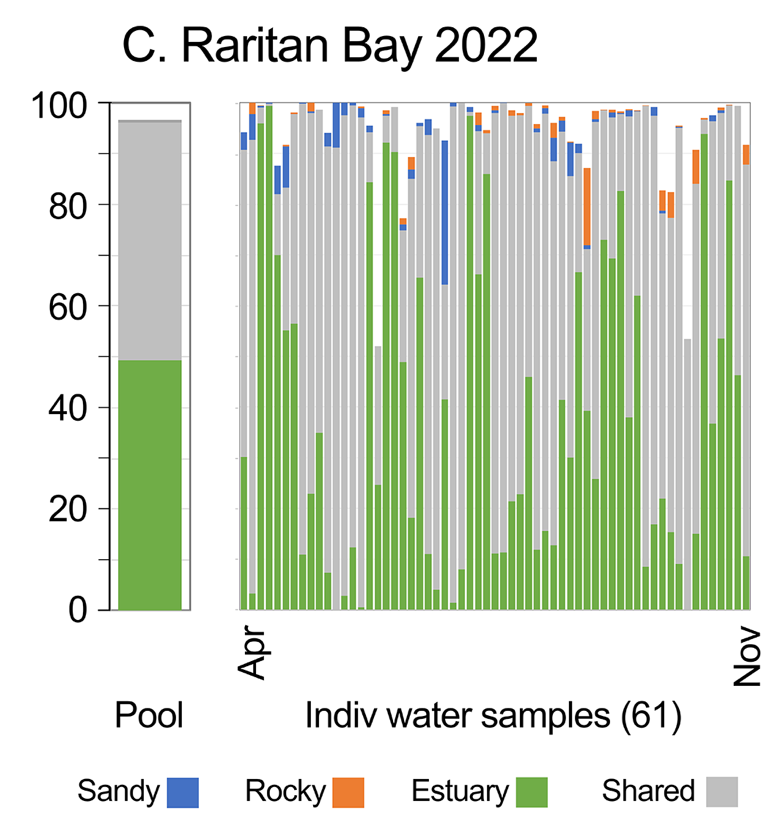
**

**S1 Fig. eDNA copies/L for Raritan Bay Seine Survey by individual water sample.** Colors indicate eDDS as derived in Fig. 2; any gap above column represents non-eDDS taxa. Similar results were obtained as compared to analysis grouped by seine day (compare to Fig 3C), specifically, 85% of individual water samples met abundance and classification criteria (85% and 90%, respectively) (source data S13 Table).
