# Supplementary material for: A potential tool for marine biogeography: eDNA-dominant fish species differ among coastal habitats and by season concordant with gear-based assessments
Source: PLoS One. 2024 Nov 11;19(11):e0313170. doi: 10.1371/journal.pone.0313170 (PMC11554088; doi:10.1371/journal.pone.0313170)
Supplement: S2 Fig — (DOCX) [file pone.0313170.s019.docx]

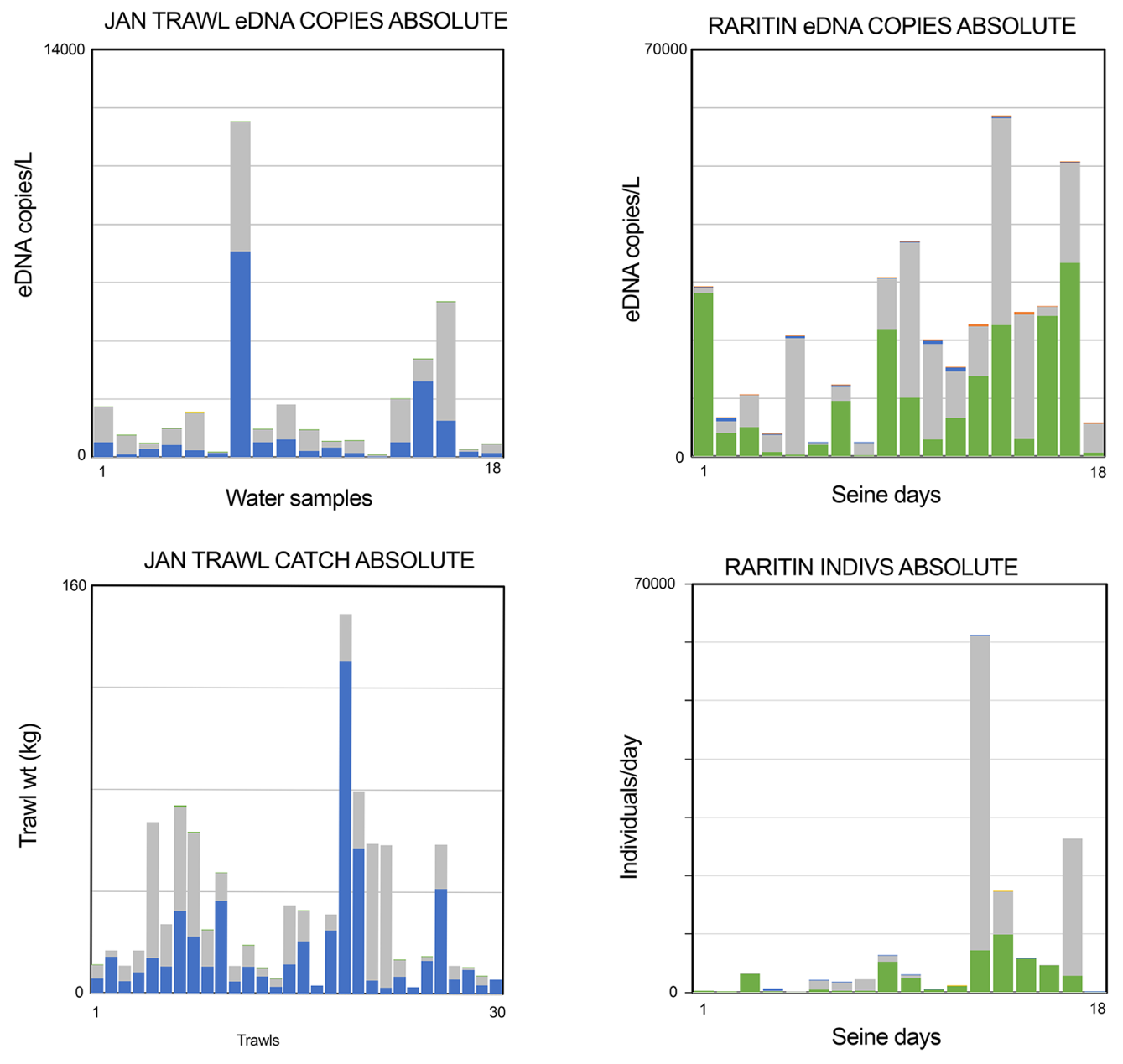


**S2 Fig. Absolute values for eDNA and capture data in January 2020 NJOTS and Raritan Bay Surveys.** Data are same as depicted in Figs. 3,4, but prior to normalizing individual observations to 100% per observation (source data Tables S4,S5,S11,S12).
