# Supplementary material for: A potential tool for marine biogeography: eDNA-dominant fish species differ among coastal habitats and by season concordant with gear-based assessments
Source: PLoS One. 2024 Nov 11;19(11):e0313170. doi: 10.1371/journal.pone.0313170 (PMC11554088; doi:10.1371/journal.pone.0313170)
Supplement: S3 Fig — (DOCX) [file pone.0313170.s020.docx]

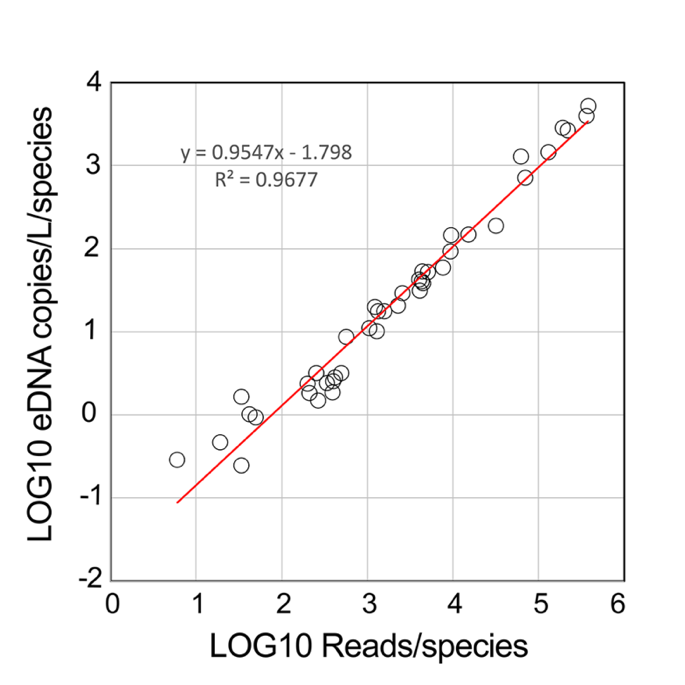


**S3 Fig. eDNA copies vs reads for NJOTS January 2020.** Each circle represents one bony fish species (n=41). Values are average eDNA copies per liter eDNA or total reads in log scale from 18 water samples. Libraries in this analysis were generated in a single MiSeq sequencing run (source data S5 Table).
